# Application of Fibronectin-Coated Microparticles for Isolation of Human iPSC-Derived MSCs

**DOI:** 10.1007/s13770-026-00822-4

**Published:** 2026-08-22

**Authors:** Jae Hyeok Jang, In Sun Hwang, Ji Seob Kim, Sunjun Lee, Min Hee Moon, Bowon Kim, Seo Jeong Kim, Jongho Choi, Young Her, Soo-Hong Lee, Byung-Hyun Cha

**Affiliations:** 1 Department of Biomedical Systems Science, College of Biomedical Science, Kangwon National University, Chuncheon-si, 24341 Republic of Korea; 2 Department of Medical Biotechnology, Dongguk University, Goyang, 10326 Republic of Korea; 3 Oral Pathology, College of Dentistry, Kangwon National University, Gangneung-si, 25457 Republic of Korea; 4 Department of Dermatology, Kangwon National University Hospital, Kangwon National University School of Medicine, Chuncheon-si, 24289 Republic of Korea

**Keywords:** iPSCs-MSCs, Fibronectin, Extracellular matrix, Three dimensional, Microparticle

## Abstract

*****BACKGROUND***:**

Bone marrow–derived MSCs (BMMSCs) are limited by availability, donor variability, and age-related decline, highlighting the need for alternative MSC sources. Induced pluripotent stem cells (iPSCs) offer a scalable solution. This study introduces a novel three-dimensional culture platform based on fibronectin (FN)-coated microparticles to efficiently derive and expand human iPSC-derived MSCs (iMSCs).

*****METHODS***:**

The system utilizes FN-coated non-porous (CytoDex) and porous (CytoPore) microparticles. FN coating efficacy on microparticle was confirmed using Rhodamine-labeled FN and confocal microscope. Base on various molecular cell biological experiment, the utilization of FN-coated CytoDex and CytoPore leverages an expanded surface area to improve iMSCs isolation. The characteristics and cell behaviors of iMSCs generated from FN-coated CytoDex and CytoPore was evaluated by immunophenotype analysis, cell proliferation, and senescence related assays. *In vivo* tissue regeneration was evaluated by microcomputed tomography and histopathological analyses.

*****RESULTS***:**

The porous microparticle CytoPore significantly enhanced FN coating efficiency, cell attachment, and proliferation compared to CytoDex. FN-coated CytoPore enabled the selective isolation of a high-purity MSC population from spontaneously differentiated iPSCs (SD-iPSCs) by day 7, confirmed by distinct morphology and mesodermal marker expression. The resulting iMSCs exhibited immunophenotypic characteristics comparable to adult MSCs, along with superior proliferative capacity, extended telomere length, and minimal senescence over 10 passages, in contrast to BMMSCs. Furthermore, iMSCs demonstrated effective *in vivo* tissue regeneration in an osteochondral defect model.

*****CONCLUSION***:**

This novel FN-coated microparticle-based 3D culture platform enables efficient, large-scale production of high-quality iMSCs and holds strong potential for clinical applications in stem cell therapeutics and regenerative medicine.

**Supplementary Information:**

The online version contains supplementary material available at https://doi.org/10.1007/s13770-026-00822-4.

## Introduction

In the field of stem cell therapy, mesenchymal stem cells (MSCs) have emerged as attractive therapeutic agents because of their ability to differentiate into mesodermal lineages such as bone, cartilage, and adipose tissues, along with their potent immunomodulatory and trophic effects [1–4]. However, conventional sources of adult tissue-derived MSCs, primarily bone marrow-derived MSCs (BMMSCs) and umbilical cord blood stem cells (UCMSCs), have several intrinsic limitations, including invasive harvesting procedures and limited cell yields, substantial donor-to-donor variability affecting cell quality and function, and an age-related decline in proliferative and differentiation capacity [5–8]. These factors significantly hinder the clinical scalability and industrial application of human adult MSC-based therapies.

To overcome these limitations and enable consistent cell quality with large-scale production potential, induced pluripotent stem cells (iPSCs) have gained attention as an alternative potential cell source for MSCs [9, 10]. iPSCs have been widely used to model human development and disease, perform drug screening, and develop cellular therapies [11]. Notably, MSCs derived from iPSCs (iMSCs) exhibit enhanced proliferative capacity, reduced senescence, and the potential to establish standardized master cell banks, thereby enabling the consistent and scalable production of therapeutic-grade cells, directly overcoming the limitations associated with adult tissue-derived MSCs [12–14]. Nevertheless, the efficient and selective isolation of functional MSCs remains technically challenging.

Despite the well-understood process of mesoderm formation during early development, the efficient translation of iMSCs into clinical-grade therapies remains a challenge. To address this, various studies have demonstrated the feasibility of MSC expansion on three-dimensional (3D) microparticles within dynamic bioreactor systems [15–17]. Compared with traditional two-dimensional (2D) culture methods, 3D microparticle-based culture systems provide a significantly increased surface-to-volume ratio that maximizes space efficiency in a controlled microenvironment [18–20]. In particular, porous microparticles offer increased surface areas, improved cell adhesion, and more uniform spatial distribution, rendering them more superior carriers for large-scale culture than non-porous microparticles such as bare microparticles[21–23]. Interestingly, previous studies in our group have shown that fibronectin (FN)-based selection enables the derivation of high-purity rejuvenated MSCs with strong self-renewal and differentiation capacities from pluripotent stem cells[24]. FN, which represents a key extracellular matrix (ECM) component, selectively promotes MSC differentiation via specific interactions with integrin α5β1, which is highly expressed on MSCs. To further enhance the scalability of such isolation and differentiation systems, diverse ECM-coated or ECM-mimicking microparticles with 3D dynamic culture platforms have garnered significant attention.

In this study, a biomimetic 3D culture system is developed using two types of microparticles—nonporous CytoDex and porous CytoPore—to selectively isolate and expand iMSCs. This study aims to compare FN-coated CytoDex-derived iMSCs (FN-Dex-iMSCs) and FN-coated CytoPore-derived iMSCs (FN-Pore-iMSCs) in terms of their pluripotent stem cell (PSC)-derived MSC characteristics, proliferation capacity, doubling time, and senescence attenuation. Additionally, this work aims to assess the multipotent differentiation ability and tissue regeneration potential through *in vitro* and *in vivo* studies using FN-Dex-iMSCs and FN-Pore-iMSCs. Ultimately, the aim is to develop a simplified and robust strategy for the scalable production of high-quality iMSCs for therapeutic applications.

## Materials and methods

### hMSC culture

Human mesenchymal stem cells (hMSCs; human bone marrow-derived mesenchymal stem cell, hBMMSCs) were cultured in 5% carbon dioxide (CO_2_) at 37 °C and maintained in high-glucose DMEM (Dulbecco’s Modified Eagle Medium; Gibco, Grand Island, NY, USA) supplemented with 10% (v/v) fetal bovine serum (FBS; Gibco) and 1% (v/v) penicillin–streptomycin (P/S; Gibco). The medium was changed every 2 *d*, and the cells were passaged at 80% confluency.

### iPSC culture

hFs-iPSC 3–1 lines were provided by the National Center for Stem Cell and Regenerative Medicine (Korea). The cell line was derived from dermal fibroblasts using Sendai virus-mediated programming. The cells were expanded on a surface coated with recombinant human truncated vitronectin (VTN-N; Gibco) in Essential Medium (Gibco). The cells were maintained at 37 °C in a 5% CO_2_ environment. The medium was changed each day.

### Preparation of microparticles for cell culture

Two physically distinct microparticles (non-porous CytoDex1 and porous CytoPore2) were purchased from Cytiva™ (Marlborough, MA, USA), and the dry particles were swollen in Ca^2+^-and Mg^2+^-free phosphate-buffered saline (PBS, 10 mg/mL particle) for > 3 h at room temperature (RT). The supernatant was decanted, and the particles were washed once with gentle agitation for a few minutes in fresh Ca^2+^-and Mg^2+^-free PBS (80 mL/g particle). The particles were sterilized by autoclaving with steam (derived from purified water; Gibco) at 121 °C for 20 min.

### Microparticle characterization

The autoclaved CytoDex1 and CytoPore2 samples were dried using a freeze dryer (MLU-9006; Mareuda), and their surface topography was analyzed by scanning electron microscopy (SEM, CX-200TM).

### Surface coating of FN on the microparticles

The microparticles were coated using an FN solution (Gibco), with loadings of 4.4 and 11 cm^2^/mg for CytoDex1 and CytoPore2, respectively. The optimized coating protocol consisted of coating the microparticles (20 mg) with the FN solution (10 mL, 0.5 μg/mL) under a 5% CO_2_ atmosphere for 90 min at 37 °C and 90 rpm.

### Confirmation of FN coating

After coating the particles with Rhodamine-conjugated FN (cytoskeleton inc., Denver, CO, USA) to verify proper coating, the particles (1 mg) were placed on a confocal dish and photographed using a confocal microscope (Nikon, ECLIPSE Ti2, motorized inverted fluorescence microscope). Additionally, the remaining rhodamine-conjugated FN was quantified using a microplate reader (BioTek; Epoch).

### Dynamic culture of iMSCs and BMMCSs with microparticles

Before dynamic culture, spinner flasks (Corning) were silicon‐treated to prevent cells from adhering to the flask walls. Cells and cells on microparticles were seeded into spinner flasks while keeping total volume at 10 mL and cultured for 2 days. The medium was kept during the process of any operations on cellular aggregates, and the sidearm caps of spinner flasks were loosened to allow for gas exchange. For the selection of iMSCs from spontaneous differentiated iPSCs, the cell generated from spontaneous differentiated iPSCs were seeded into spinner flask (Corning) at 0 rpm for 12 h under the above media conditions of hBMMSCs. After 12 h of the iMSCs-FN on microparticle selection, iMSCs were cultured and maintained into spinner flask (Corning) at 45 rpm. The media was changed every 2 days. Also, BMMSCs were seeded in spinner flask at 0 rpm for 12 h under the above media conditions of hBMMSCs. After 12 h, hBMMSCs were cultured and maintained at 45 rpm. Also, the media was changed every 2 days.

### Assessment of iMSC attachment to FN-coated microparticles

SD-iPSCs were seeded onto CytoDex or CytoPore, and incubation for 12 h. The supernatant containing non-adherent cells was passed through a 100 μm cell strainer to retain the microparticles, and the filtrate containing non-adherent cells was collected. The number of unattached cells was determined using a cell counter. The percentage of cell attachment to CytoDex or Cytopore was calculated by subtracting the unattached cells from the initial cell concentration. Cell adhesion efficiency (%) = [(initial seeded viable cells − unattached viable cells) / initial seeded viable cells] * 100.

### Fluorescence-based evaluation of cell expansion

Cell expansion of FN-Dex-iMSCs and FN-Pore-iMSCs was determined using a viability kit assay (Invitrogen, MA, USA). iMSCs were seeded onto FN-coated CytoDex or CytoPore microparticles and subjected to Live/Dead staining on days 1, 3, and 5 after attachment. After a brief incubation of 15 min at 20–25 °C, the cells were visualized using a fluorescence microscope. The fluorescence intensity of FN-Dex-iMSCs and FN-Pore-iMSCs was quantified using ImageJ software to evaluate cell expansion capacity.

#### FN Matrix binding-based separation of MSCs from spontaneously differentiated hiPSCs (SD-iPSCs) using microparticles

Small clumps of hiPSCs were transferred onto a surface coated with VTN-N and cultured in Essential Medium 8 (Gibco) under humidified air containing 5% CO_2_ at 37 °C. The medium was replaced daily until the hiPSC colonies reached 30% confluency. For the spontaneous differentiation of iPSCs, the cells were incubated in a spontaneous differentiation medium comprising DMEM/F12 supplemented with 10% FBS (Gibco), 1% Non-Essential Amino Acids (NEAA; Gibco), and 1% P/S for 7 *d*. The cells were treated with 1% DMSO (Sigma-Aldrich, St. Louis, MO, USA) for 12 h during the early stages of spontaneous differentiation. To separate the MSC-like cells from spontaneously differentiated cells via FN-mediated binding, the cells were dissociated into single cells and seeded onto FN-coated particles. After incubation for 12 h at 37 °C, nonadherent cells were removed by rinsing with PBS. The medium was changed every 2 *d*. Adherent cells were retained and subcultured every 3–4 *d* until passage 3.

#### Flowcytometry analysis

Cell-surface antigens were evaluated using flow cytometric analysis. The cells were dissociated with 0.25% Trypsin–EDTA (1 ×), washed with PBS, fixed using 4% paraformaldehyde, and blocked with FACS buffer (Invitrogen™). The cells were then stained with antibodies against human leukocyte antigen–DR isotype conjugated to APC, and CD44 CD73, CD90, and CD105 conjugated to FITC for 30 min in the dark at 4 °C. Cell immunotypes were determined using a FACSymphony A3 (BD Biosciences, Franklin Lakesm, NJ, USA) and the percentage of expressed cell surface antigens was calculated for 100,000 gated cell events.

#### Cell proliferation and population doubling time assays

To determine the cumulative cell numbers from passages 3 to 7, the cells at each passage were trypsinized from individual wells, transferred to low-glucose DMEM containing 10% FBS to neutralize trypsin, and counted using a hemocytometer C-Chip (NanoEntek inc., Seoul, Korea). To evaluate the population doubling time (PDT), the human BMMSCs (ATCC, Manassasm VA, USA) and iMSCs obtained after passage 3 were counted and cultured at a starting density of 7 × 10^3^ cells per well in 6-well plates. At 80% confluence, the cells were trypsinized, counted, and re-plated. The PDT evaluation was repeated three times starting from passage 3 for each group of cells, and the average cell number was used for the final calculations. This process continued up to passage seven (minimum 28 *d*), and the *in vitro* doubling time was calculated using an exponential curve equation, DT = (t—t0) * ln(2) / ln(N / N0), where t—t0 represents the culture time, N is the final cell number, and N0 is the initial cell number.

#### Measurement of the telomere length

The relative telomere length was quantified by quantitative real-time polymerase chain reaction (qPCR) as the ratio of telomere repeat amplification products to a single-copy gene product (IFNB1). Genomic DNA (gDNA) was isolated using a genomic DNA kit (Bioneer, Daejeon, Korea) and quantified using a Nanodrop spectrophotometer. gDNA (5 ng) was employed for qPCR using TOPreal™ qPCR 2X PreMIX (SYBR Green with low ROX; Enzynomics, Daejeon, Korea). The primers employed in the current study were as follows:

- TEL-F 5-CGGTTTGTTTGGGTTTGGGTTTGGGTTTGGGTTTGGGTT-3.
- TEL-R 5-GGCTTGCCTTACCCTTACCCTTACCCTTACCCTTACCCT-3.
- IFNB1-F 5-GGTTACCTCCGAAACTGAAGA-3.
- IFNB1-R 5-CCTTTCATATGCAGTACATTAGCC-3.

#### β-Galactosidase staining

BMMSCs and iMSCs were seeded into 6-well plates, and the MSC culture medium was eluted when the cell density reached 80%. Subsequently, the plate was rinsed with 1 × PBS and 1 × fixative solution (1 mL) from the Senescence β-Galactosidase Staining Kit (Cell Signaling Technology) was added. After fixation for 15 min, the plate was rinsed twice with 1 × PBS. A β-galactosidase staining solution was prepared according to the manufacturer protocol, and the cells were stained with an aliquot (1 mL) of this staining solution. The plate was incubated at 37 °C overnight in a dry incubator to allow a blue color to develop, after which the cells were examined under a microscope (200 × total magnification).

#### *In Vivo* osteochondral defect model

Animal surgeries were performed according to protocols approved by the Dongguk University Institutional Animal Care and Use Committee for the care and use of laboratory animals (Approval number # IACUC-2024–036-1). Healthy male Sprague Dawley rats (12 weeks old and weighing 300–350 g) were used for the study. Animals were anesthetized with a mixture of tiletamine hydrochloride, zolazepam hydrochloride (Zoletil, 50 mg kg^−1^, Virbac Laboratories, Carros, France), and xylazine (Rompun, 10 mg kg^−1^, Bayer, Seoul, South Korea). During surgery, each animal was administered an intraperitoneal injection of normal saline to account for fluid loss. Osteochondral defect generation and subsequent transplantation of BMMSCs, FN-Dex-iMSCs, and FN-Pore-iMSCs (1 × 10^7^ cells per site, respectively) in rats were performed as follows: a lateral parapatellar longitudinal incision was made to expose the knee joint during surgery. The synovial capsule was incised and the trochlear groove was exposed after medial luxation of the patella. With the knee maximally flexed, a defect 2 mm in diameter and 2 mm in depth was created in the center of the groove using a dental drill. All debris was removed from the defect with a curette and irrigation. Depending on the experimental group, the defect was left untreated or treated with BMMSCs, FN-Dex-iMSCs, and FN-Pore-iMSCs in 2% hyaluronic acid (Sigma-Aldrich). The patella was physically relocated and the joint capsule and subcutaneous tissue were closed.

#### Microcomputed tomography analysis

The microstructural morphology of the lumbar spines was evaluated using a SkyScan-1076 micro-CT device (SkyScan, Kontich, Belgium) at 8 weeks after implantation. The X-ray source was set to a voxel size of 18 mm at 40 keV and 250 mA. The exposure time was 520 ms with a frame average of 3. X-ray beam filtration with 1 mm aluminum was used. Data were recorded at rotation step intervals of 0.4° until 180°. Image slices were reconstructed using the NRecon software (Skyscan) based on the Feldkamp algorithm and by applying a correction for the beam. And, the new bone mass was isolated from the native bone through a manually drawn region of interest (ROI). The outline of the ROIs was manually drawn using CT-Analyzer 3D data analysis software (Skyscan) and care was taken not to select outgrowing mineralized osteophytes.

#### Sample preparation for histological analysis

Rat femurs collected after euthanasia were fixed in 10% neutral-buffered formalin for 3 days and subsequently decalcified in 10% EDTA until sufficiently softened for sectioning. After gross trimming to isolate the experimental region, tissues were processed and embedded in paraffin to prepare blocks for histological analysis.

#### Hematoxylin and Eosin (H&E) staining

Paraffin-embedded specimens were sectioned at 3 μm, deparaffinized, and rehydrated through a graded ethanol series (100–70%). After rinsing in running tap water, sections were stained with Mayer’s hematoxylin for 10 min, followed by an additional 10 min wash. Slides were counterstained with 1% alcoholic eosin Y for 3 min, dehydrated with 70% ethanol, 95% ethanol, and two changes of 100% ethanol, and mounted using Permount™ Mounting Medium (Electron Microscopy Sciences, Hatfield, PA, USA). Stained sections were imaged using a PANNORAMIC 250 Flash III digital slide scanner (3DHISTECH Ltd., Budapest, Hungary).

#### Safranin O/Fast green staining and quantification

Sections were deparaffinized, rehydrated to distilled water, and stained with Weigert’s iron hematoxylin working solution for 10 min, followed by a 10 min wash. Samples were then stained with 1% fast green FCF for 5 min and briefly rinsed with 1% acetic acid for 10–15 s. Counterstaining was performed with 0.1% safranin O for 5 min. Slides were dehydrated and cleared using 95% ethanol, absolute ethanol, and xylene (two changes each, 2 min per change), mounted with coverslips, and imaged using a PANNORAMIC 250 Flash III scanner. The Fast Green-positive area was quantified using ImageJ software to evaluate bone matrix area (mm^2^).

#### Statistical analysis

Numerical data are presented as the mean ± standard deviation. All statistical analyses were performed using GraphPad Prism version 10.4.1 (San Diego, CA, USA). The unpaired Student’s t-test was used to compare two groups, while one-way or two-way analysis of variance (ANOVA) was used to compare multiple groups. The outcomes were analyzed using Tukey’s post-hoc test. A *p* value of < 0.05 was considered statistically significant.

## Results and discussion

### Selective isolation of iMSCs from spontaneously differentiated iPSCs using FN-coated microparticles

**Fig. 1:**
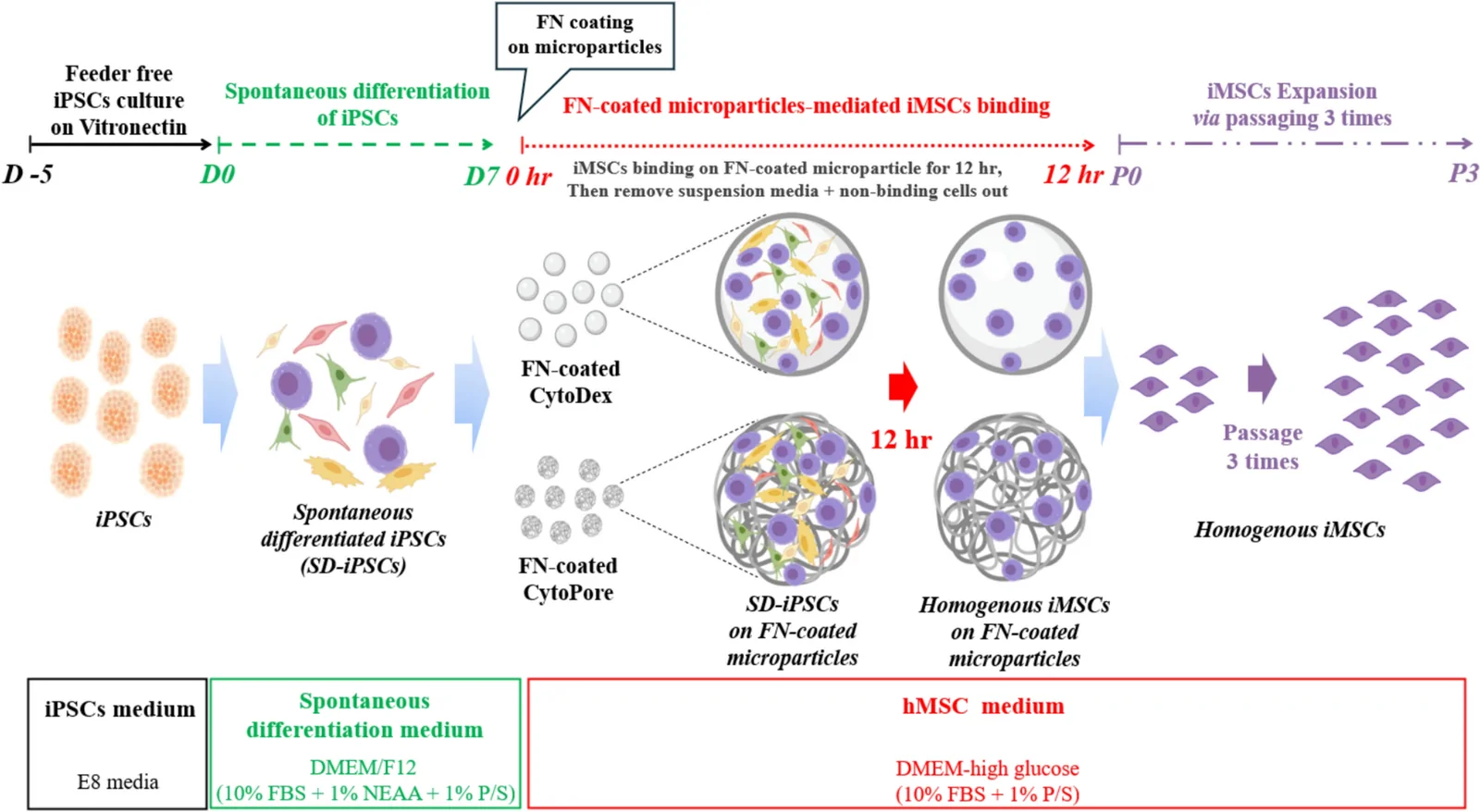
Schematic illustrating fibronectin (FN)-coated microparticle–mediated selection of hiPSC-derived MSCs (iMSCs). Microparticle and FN based isolation designed for large-scale MSC harvesting in limited space by spontaneous differentiation of iPSCs, seeding onto FN-coated CytoDex and CytoPore) and subsequent washing to selectively retain and culture only FN-adherent MSC progenitors on the microparticles

The increasing demand for MSCs in regenerative medicine necessitates the development of a reliable and scalable production platform to overcome the intrinsic limitations of BMMSCs, including their limited proliferative capacity, donor heterogeneity, and senescence [25, 26]. To address these intrinsic limitations, research trends have shifted from iMSCs to the development of an efficient and scalable platform for generating clinical-grade MSCs. To expand on previous FN-based strategies for the development of efficient MSC isolation techniques, a bioinspired 3D culture platform using FN-coated microparticles was developed to isolate iMSCs based on integrin-mediated cell adhesion. Accordingly, as shown in Fig. 1, a novel isolation protocol was designed by combining spontaneously differentiating human iPSCs (SD-iPSCs) and an ECM-mediated binding selection step via FN-coated microparticles, which differs from cytokine cocktail-based differentiation protocols [27, 28]. Before performing the spontaneous differentiation of hiPSCs, their typical colony-based pluripotency characteristics were observed on day 5 (Fig. S1). To determine the optimal differentiation period of SD-iPSCs, temporal changes in the expression of three germ layer-specific genes (endoderm, mesoderm, and ectoderm) were analyzed during the spontaneous differentiation of the undifferentiated hiPSCs (Fig. S2A). Similar to our previous reports [24], it was found that mesoderm lineage-related markers, such as *Slug/Snail* and *Brachy*, were highly expressed on day 7 compared to their expression levels on days 5 and 9. Additionally, the expression levels of other endoderm- and ectoderm-related markers were lower than those of *Slug/Snail* and *Brachy* on day 7. Human iPSCs were therefore cultured in a vitronectin-based feeder-free system for 7 *d* to promote spontaneous differentiation. Furthermore, a heterogeneous morphology and loss of the typical undifferentiated iPSC colony morphology were observed on day 7 of spontaneous differentiation (Fig. S2B). FACS analysis was performed to quantify the MSC surface marker distribution in SD-hiPSCs, revealing that 23.90% of the population was double positive for CD90^+^ and CD105^+^ (Fig. S2C). As previously reported, SD-iPSCs generate a heterogeneous population containing lineage-differentiated cells as well as residual undifferentiated iPSCs [4, 29, 30]. To enable FN-mediated binding to the microparticles, single SD-iPSCs obtained by conventional enzyme dissociation were transferred onto FN-coated microparticles and incubated for 12 h. Following FN-mediated binding, nonadherent cells were removed. For *in vitro* expansion and stabilization of differentiation, the selected cells were subsequently passaged up to passage 3 [31, 32].

### Morphological characterization and verification of cytodex and cytopore microparticles after FN coating

**Fig. 2:**
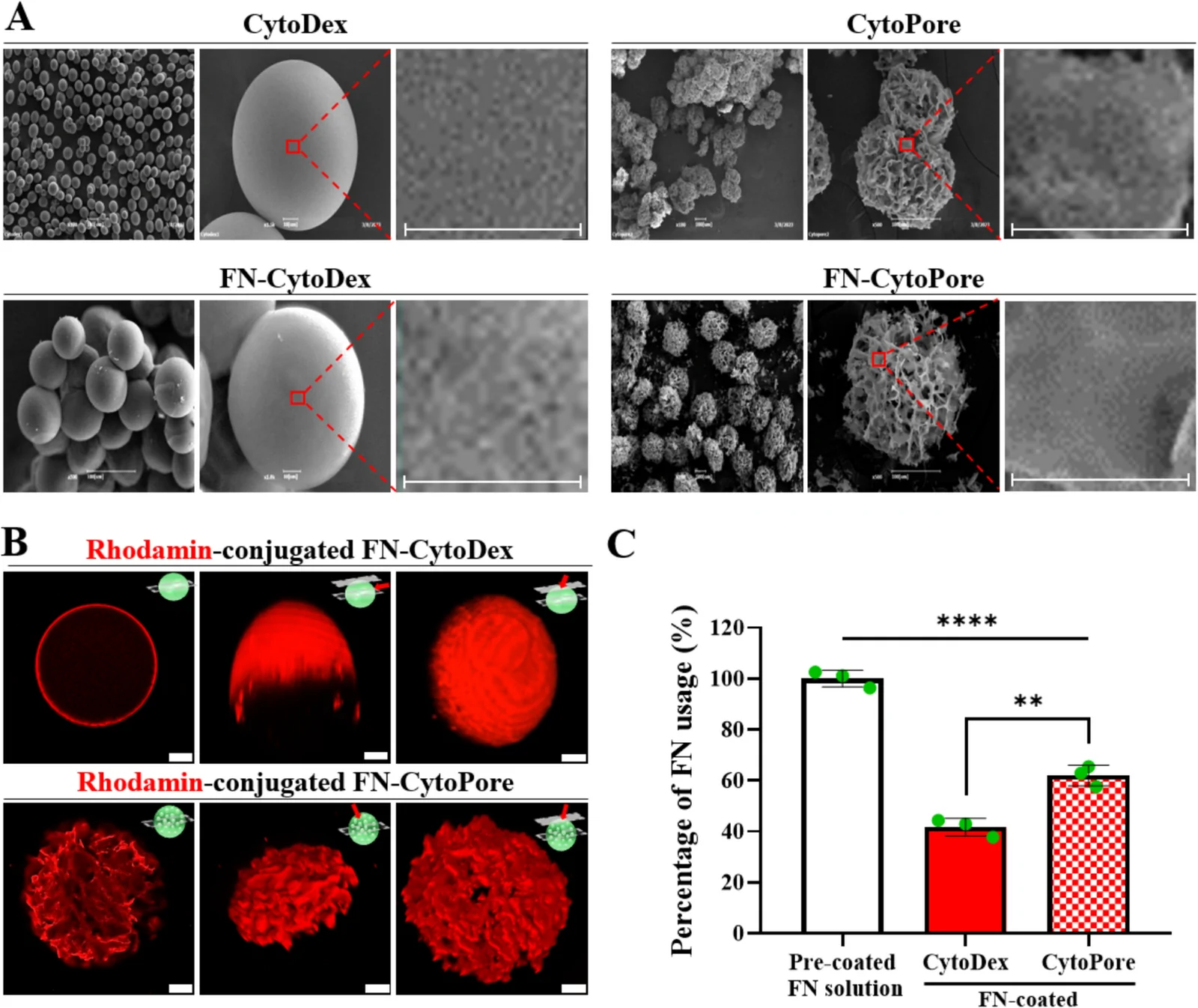
Morphological characterization and verification of CytoDex and CytoPore microparticles after FN coating. **A** Scanning electron microscope image of none-coated (top) and FN-coated (bottom) CytoDex and CytoPore microparticles (scale bar = 10 µm). **B** Confocal image of rhodamine-conjugated FN–coated CytoDex and CytoPore microparticles (scale bar = 40 µm). **C** Quantification of FN loading on microparticles after coating. Data are presented as mean ± s.d, n = 3. Statistical significance is indicated as ** *p* < 0.01, **** p < 0.0001

Specifically, two commercially available microparticles with distinct structural features were employed, namely CytoDex, composed of dextran with a smooth and non-porous surface and a diameter range of 131–220 μm, and CytoPore, composed of cellulose with a spiky, rough, and porous surface and a diameter range of 200–280 μm (data from Cytiva™). Compared with CytoDex, CytoPore, with its multicavity surface, demonstrated the highest cell attachment and growth rate (4.4 cm^2^/mg for CytoDex vs. 11 cm^2^/mg for CytoPore). These structural advantages may provide a larger effective surface area for cell adhesion and expansion, supporting the efficient growth of adherent cells in a 3D dynamic culture environment [33, 34]. As shown in Fig. 2A, the surface characteristics of the CytoDex and CytoPore microparticles were confirmed by SEM, revealing distinct surface morphologies that included nonporous and porous structures. After the post-FN coating process on both types of microparticles, the gross surface morphologies of FN-CytoDex and FN-CytoPore were not visibly altered. Additionally, to visualize and confirm the FN coating on both microparticles, Rhodamine-labeled FN was applied to the FN-CytoDex and FN-CytoPore microcarriers. As shown in Fig. 2B, fluorescence imaging revealed a uniform and continuous FN coating on the surface of CytoDex. In contrast, CytoPore exhibited intense fluorescence distributed throughout its porous matrix, indicating substantial FN penetration and internal coating, which is likely attributable to its open and interconnected porous architecture. Rhodamine-labeled FN visualization confirmed deep matrix penetration and internal coating in CytoPore, enabling a higher density of bioactive adhesion sites [35, 36]. This structural advantage may support an increased density of biologically active adhesion sites, which could contribute to improved selectivity and efficiency in capturing MSCs from SD hiPSCs. Moreover, as shown in Fig. 2C, quantitative analysis of FN adsorption after the coating process revealed that CytoPore exhibited a significantly higher FN coating efficiency (61.94 ± 4.00%) than CytoDex (41.69 ± 3.48%), while the pre-coated FN solution served as a positive reference control. These results demonstrate the superior FN-binding capacity of CytoPore and highlight its potential for establishing a favorable microenvironment for the selective adhesion and expansion of iMSCs. As FN coating was achieved by physical adsorption, the stability of FN on the microparticle may be influenced by culture conditions.

### Cell adhesion and expansion of iMSCs on FN-coated cytodex and cytopore

**Fig. 3:**
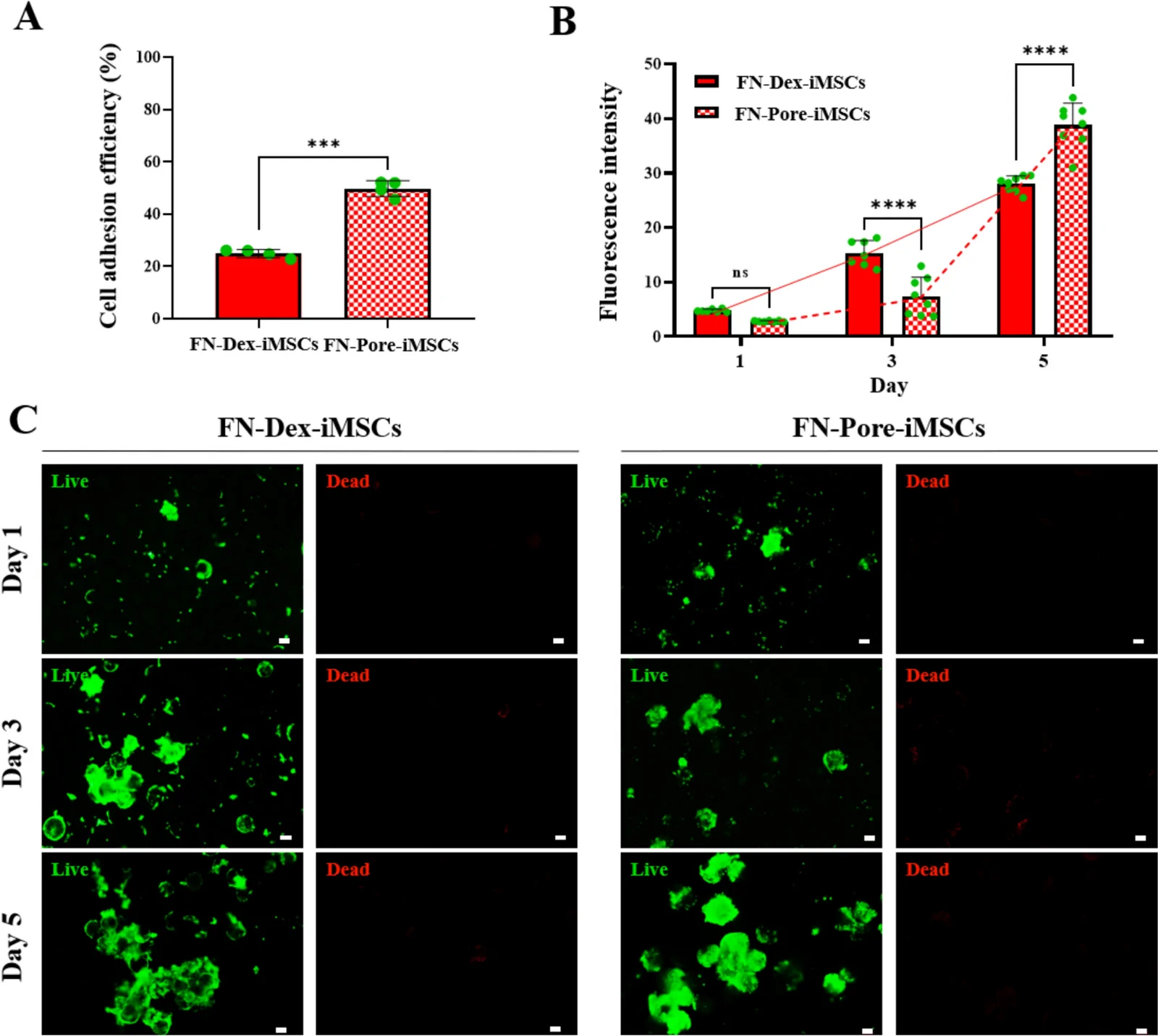
Cell adhesion and expansion of iMSCs on FN-coated CytoDex and CytoPore. **A** Cell-adhesion efficiency during FN-coated microparticle-based isolation from SD-iPSCs. Data are presented as mean ± s.d, n = 4. Statistical significance was set at *** p < 0.001. **B** FN-coated microparticle ROI-based quantification of cell expansion. n indicates the number of microparticles. Data are presented as mean ± s.d, n = 8. Statistical significance was set at ns, not significant, ****p < 0.0001. **C** Live/dead staining of iMSCs cultured on FN-coated CytoDex and CytoPore (Calcein AM in green, and BOBO-3 Iodide in red) (scale bar = 100 µm)

To confirm the selection platform for human iMSCs, the cytocompatibility and expansion efficiency of iMSCs were evaluated using FN-coated CytoDex and CytoPore (FN-Dex-iMSCs and FN-Pore-iMSCs, respectively). After 12 h of cell binding via FN-coated microparticles, the FN-Pore-iMSCs (49.76 ± 3.04%) showed a significantly higher level of cell adhesion efficiency compared to the FN-Dex-iMSCs (24.88 ± 1.52%) (Fig. 3A). FN-coated CytoPore exhibited significantly greater cell adhesion efficiency than FN-coated CytoDex, indicating that porous cellulose-based microcarriers provide a more favorable surface for selective iMSC capture. To validate the biocompatibility and proliferative capacity of the FN-coated microparticles, live/dead assays were performed over a 5-d culture period (Fig. 3B and C). Quantitative analysis of the fluorescence intensity of randomly selected particles confirmed these observations. Specifically, green fluorescence intensity, which reflects the viable cell density, increased significantly from day 1 to 5 in both FN-Dex-iMSCs and FN-Pore-iMSCs. On day 1, both FN-Dex-iMSCs and FN-Pore-iMSCs exhibited low levels of green fluorescence, without any observable red fluorescence. From day 1 to day 5, a progressive increase in viable cells was observed for both microparticle types, with enhanced cell aggregation evident by day 3 and extensive cell–cell interactions formed by day 5. Notably, on day 5, the FN-Pore-iMSCs supported more extensive cell proliferation and aggregation, suggesting superior performance in sustaining cell growth via the porous CytoPore architecture. These results demonstrate that porous-based CytoPore microcarriers support greater initial iMSC attachment and viability across five days of culture than non-porous CytoDex, suggesting that microstructural porosity enhances not only the surface area but also the biophysical presentation of ECM ligands, ultimately promoting selective MSC capture [24]. However, this study was limited to two commercial microcarrier systems, CytoDex and CytoPore, and was not designed as a GMP-compatible manufacturing. Therefore, additional studies using diverse microcarriers and bioreactor-based culture systems will be required to confirm FN-coating reproducibility, cell expansion efficiency, cell recovery and detachment, and residual microcarrier removal from the final cell product.

### Phenotype and biological features of iMSCs via selection of FN-coated cytodex and cytopore

**Fig. 4:**
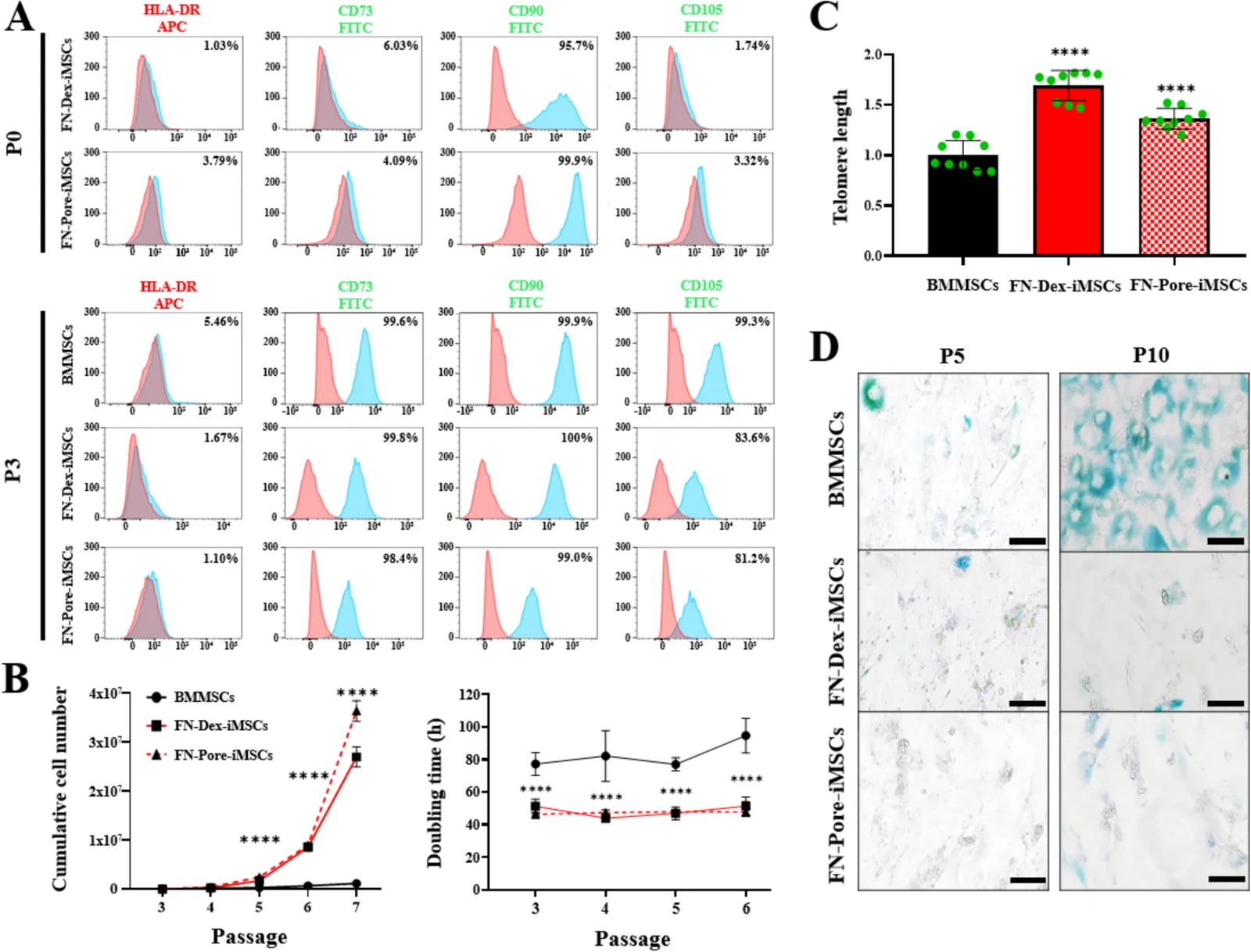
Phenotype and biological features of iMSCs via selection of FN-coated CytoDex and CytoPore **A** Surface antigen flow-cytometric profiling at P0 and P3 FN-Cytodex-derived iMSCs (FN-Dex-iMSCs) and FN-CytoPore-derived iMSCs (FN-Pore-iMSCs). CD73, CD90, and CD105 are positive MSC markers and HLA-DR are negative MSC markers. **B** Cumulative each cell number during P3 to P7 passaging. Cumulative cell number was calculated as the running sum of viable harvested cells, while starting from 70,000 cells per condition. Data are presented as mean ± s.d, n = 3. Statistical significance was set at **** *p* < 0.0001. (left graph) Viable cell yield-based doubling time analysis across passages (P3-P6). Data are presented as mean ± s.d, n = 3. Statistical significance was set at **** *p* < 0.0001. (right graph) **C** Quantification of telomere length by qPCR. Values were normalized to BMMSCs and are presented as mean ± s.d, n = 9. Statistical significance was set at ****p < 0.0001. **D** Representative bright-field images of senescence-associated β-galactosidase (SA-β-gal) staining at passage 5 (P5) and passage 10 (P10) (scale bar = 100 µm)

To assess the functional identity of iMSCs isolated using FN-coated CytoDex and CytoPore, the expression of principal MSC surface markers was first analyzed using flow cytometry. At passage 0 (P0), FN-Dex-iMSCs and FN-Pore-iMSCs exhibited partial MSC marker profiles, with low expression of CD73 and CD105, and high CD90 positivity, while retaining minimal expression of HLA-DR, a negative MSC marker (Fig. 4A). This profile suggests that the isolated cells possessed an early MSC progenitor-like status. Upon expansion to passage 3 (P3), both iMSC populations matured into fully defined MSCs, showing surface marker profiles comparable to those of BMMSCs, namely high CD73, CD90, and CD105 expression (> 80%) and sustained HLA-DR negativity [24, 30, 37]. This transition suggests that FN-based selection not only captures mesodermal progenitors but also supports their maturation under defined culture conditions. These results confirmed that FN-coated microparticles enable the selective isolation and maturation of iPSC-derived MSCs toward an MSC-like phenotype.

Subsequently, the proliferative performance across passages was evaluated. Both FN-Dex-iMSCs and FN-Pore-iMSCs demonstrated significantly higher cumulative cell numbers than BMMSCs during continuous culture from passages 3 to 7 (Fig. 4B, left). Previous studies have reported that hPSC-derived MSCs have significant advantages in generating sufficient cell quantities with high proliferative capabilities for clinical applications [38–40]. Furthermore, both FN-Dex-iMSCs and FN-Pore-iMSCs maintained consistent doubling times across passages, whereas BMMSCs exhibited progressively increasing doubling times, reflecting a declining proliferation rate over time (Fig. 4B, right). On average, the doubling times of FN-Dex-iMSCs (48 ± 3.0 h) and FN-Pore-iMSCs (47 ± 0.5 h) were markedly shorter than those of BMMSCs (80 ± 6.0 h), highlighting their superior proliferative capacity [41–43].

To assess cellular aging, telomere length analysis was performed using qPCR, revealing that both the FN-Dex-iMSCs and FN-Pore-iMSCs (1.692 ± 0.1149 and 1.363 ± 0.1026, respectively) retained significantly longer telomere lengths than the BMMSCs (1.000 ± 0.1465), further supporting their enhanced replicative potential (Fig. 4C). In the same context, senescence-associated β-galactosidase (SA-β-gal) staining was performed at passage 5 and 10. BMMSCs showed a marked increase in senescent cells at passage 10, whereas both the FN-Dex-iMSC and FN-Pore-iMSC populations exhibited minimal senescence-associated staining, even after prolonged expansion (Fig. 4D), indicating resistance to culture-induced senescence. These results demonstrate that during long-term culture, FN-Dex-iMSCs and FN-Pore-iMSCs delay the onset of senescence more effectively than adult BMMSCs [44–46]. Additionally, the isolation potential of BMMSCs on both FN-coated CytoDex and CytoPore was confirmed (Fig. S3), suggesting that this isolation method could be applied to fully differentiated MSCs. These findings revealed that FN-coated microparticles represent a useful biomaterial for isolating and enriching CD90^+^CD105^+^ cells from SD-iPSCs via interactions between FN and integrin α5β1 on cells [24, 47, 48]. Overall, these results demonstrate that FN-coated CytoPore microparticles provide a superior microenvironment for efficient isolation, viability maintenance, and expansion of iMSCs, outperforming conventional smooth-surfaced carriers, such as CytoDex. Collectively, these advantages overcome the limited *in vitro* expansion potential of BMMSCs, underscoring the promise of FN-microparticle-derived iMSCs as a scalable and sustainable cell source for regenerative therapies.

### Therapeutic effects of iMSCs (passage 3) via FN-coated cytodex and cytopore for osteochondral defect model

**Fig. 5:**
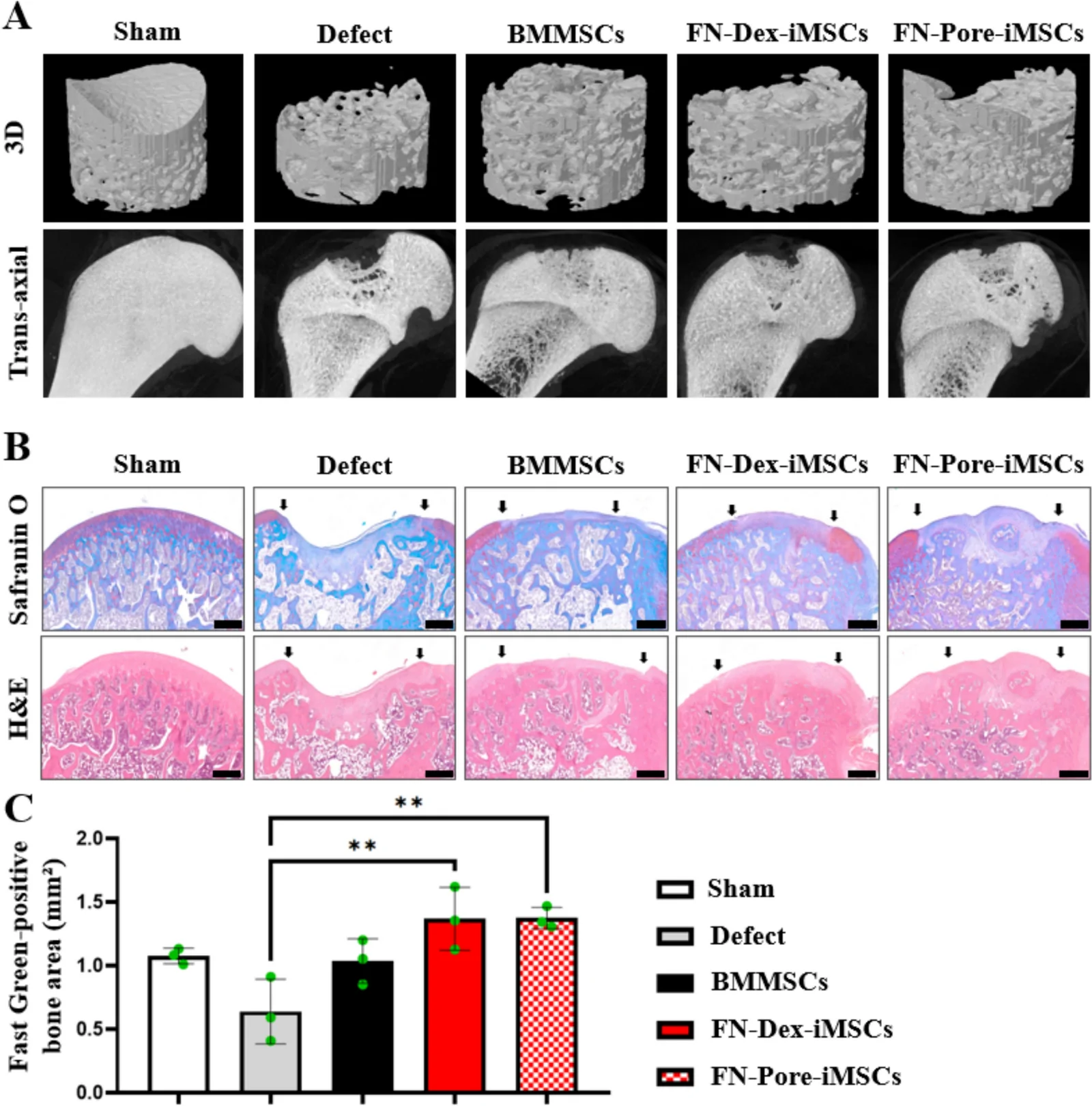
Therapeutic effects of iMSCs (passage 3) via FN-coated CytoDex and CytoPore for osteochondral defect model **A** Representative 3D micro-CT images (3D reconstructed image and trans-asxial cross-section) of the osteochondral defect at 8 weeks post-treatment (Sham, Defect, BMMSCs, FN-Dex-iMSCs, and FN-Pore-iMSCs). **B** Representative histological images (Safranin O and H&E staining) of the osteochondral defect at 8 weeks post-treatment (Sham, Defect, BMMSCs, FN-Dex-iMSCs, and FN-Pore-iMSCs). (scale bar = 500 µm). **C** Fast Green-positive bone area was quantified within the region indicated by two arrows in Safranin O/Fast Green-stained sections and expressed as area (mm^2^). Data are presented as mean ± s.d, n = 3. Statistical significance is indicated as ** *p* < 0.01

Before confirming the *in vivo* differentiation potential, the *in vitro* multilineage differentiation capacity of FN-Dex-iMSCs and FN-Pore-iMSCs was evaluated (Fig. S4). After 2 and 3 weeks of differentiation, both FN-Dex-iMSCs and FN-Pore-iMSCs demonstrated *in vitro* multilineage differentiation potential, as confirmed by histological staining [49, 50]. To evaluate the *in vivo* tissue regeneration potential of iMSCs generated from both types of FN-coated microparticles, a critically sized osteochondral defect model was employed [51–53]. As shown in Fig. 5A, micro-computed tomography analysis revealed substantial bone formation in the BMMSC, FN-Dex-iMSC, and FN-Pore-iMSC groups compared to those observed in the sham and defect groups. The 3D and transaxial images showed that all cell-injected groups resulted in more continuous and denser trabecular-like bone structures than those found in the defect group. Additionally, cartilage regeneration was assessed histologically using Hematoxylin and Eosin and Safranin O staining (Fig. 5B). The defect group exhibited extensive proteoglycan depletion, as observed by Safranin O staining. In contrast, the FN-Dex-iMSC and FN-Pore-iMSC groups showed abundant cellularity and cartilage architecture, resembling that of the sham group. Compared with the defect and BMMSC groups, the FN-Dex-iMSC and FN-Pore-iMSC groups demonstrated relatively well-organized and abundant proteoglycans. These effects likely resulted from a combination of paracrine signaling (e.g., VEGF, IGF-1, and TGF-β) and direct contribution to osteogenic matrix deposition, both of which are enhanced in iMSCs derived under optimized integrin–FN interaction conditions [54–57]. Although secretome profiling was beyond the scope of this study, the observed osteochondral tissue regeneration suggests that iMSCs generated from FN-coated microparticles possess both trophic and differentiation capacities, which are critical for osteochondral repair. These results confirm that iMSCs isolated from FN-coated microparticles possess superior regenerative potential *in vivo*, supporting their applicability in diverse tissue engineering and stem cell therapy.

In conclusion, this study presented a bioinspired 3D culture platform that utilizes FN-coated porous microcarriers to selectively isolate and expand iPSC-derived MSCs with enhanced proliferative and regenerative capacities. This system provides both biochemical and mechanical cues essential for early lineage commitment, yielding high-purity senescence-resistant MSCs suitable for *in vivo* therapeutic applications. Our findings support the clinical translation of iPSC-MSCs using engineered microenvironments, marking a significant step toward scalable and reproducible stem cell manufacturing for regenerative medicine. Future studies will aim to evaluate the transcriptomic and proteomic profiles of iMSC populations via FN-coated microparticles, as well as optimizing bioreactor-based dynamic culture systems for scalable clinical-grade production. Finally, the integration of stimuli-responsive or gradient-functionalized microcarriers will be investigated to further enhance lineage specificity and therapeutic potency.

## Supplementary Information

Below is the link to the electronic supplementary material.

Supplementary file1 (DOCX 1603 KB)

## Data Availability

The datasets used and/or analyzed during the present study are available from the corresponding author on reasonable request.
