# Supplementary material for: Application of Fibronectin-Coated Microparticles for Isolation of Human iPSC-Derived MSCs
Source: Tissue Eng Regen Med. 2026 Aug 22;23(7):1027–40. doi: 10.1007/s13770-026-00822-4 (PMC13620072; doi:10.1007/s13770-026-00822-4)
Supplement: Supplementary file 1 [file 13770_2026_822_MOESM1_ESM.docx]

Supplementary Material

**Application of Fibronectin-coated Microparticle for** **Isolation of Human iPSC-derived MSCs**

**Materials and Methods**

**Alkaline Phosphatase Staining**

Alkaline phosphatase (ALP) staining was performed using an ALP staining kit (Purple) (Abcam). iPSCs were fixed in the fixing solution for 2 min at RT and the cells were washed with PBST. Subsequently, the ALP staining solution was added in the dark and allowed to stand at RT for 15–30 min. Images were obtained randomly using a microscope (ECLIPSE TS2-FL; Nikon, Tokyo, Japan).

**Immunocytochemistry**

Samples were fixed with 4% paraformaldehyde (Biosesang, Seongnam, Korea), permeabilized with 0.1% Tween 20 (Sigma-Aldrich), and blocked with a mixture of bovine serum albumin (BSA; Sigma-Aldrich) diluted in PBS. Samples were incubated with primary antibodies (Oct4, Abcam, Cambridge, UK, 1:200; Sox2, Abcam, 1:1000; Nanog, Abcam, 1:250; and SSEA-4, Abcam, 1:1000) for 1 h at RT. The samples were subsequently stained with fluorescently labeled secondary antibodies (Alexa Fluor-594 conjugated goat anti-rabbit (Abcam, 1:500) and Alexa Fluor-488 conjugated goats anti-mouse (Abcam, 1:500)) for 1 h at RT. The samples were then washed and mounted for imaging using a fluorescence microscope (ECLIPSE TS2-FL; Nikon, Tokyo, Japan).

**RNA Isolation and Quantitative Real-Time Polymerase Chain Reaction (qPCR)**

Total RNA was isolated using the TRIzol reagent (Invitrogen) according to the manufacturer’s instructions. mRNA was reverse-transcribed into complementary DNA (cDNA) using a cDNA synthesis kit (Bio-Rad Laboratories Inc., Herculesm, CA, USA). qPCR analysis was performed using TOPreal^TM^ qPCR 2X PreMIX (SYBR Green with low ROX) (Enzynomics) on a Quant Studio^TM^ 1 System (Applied Biosystems; Thermo Fisher Scientific Inc., Waltham, MA, USA) according to the manufacturer’s instructions.

**Confrimation of multi-lineage differentiation of MSCs**

To confirm the multipotent properties of iPSCs‐MSCs, we induced adipogenic, chondrogenic, and osteogenic differentiation in iPSCs‐MSCs, using a chemically defined medium at passage 4. The osteogenic differentiation medium consisted of DMEM/High media with 10% FBS, 1% P/S, 10 mM glycerol‐2‐phosphate (Sigma-Aldrich), 50 μg/mL ascorbic acid, 100 nM dexamethasone (Sigma), and 1% Glutamax (Gibco). The adipogenic differentiation medium was prepared using DMEM/High media containing 10% FBS, 1% P/S, 10 μg/mL insulin (Sigma-Aldrich), 500 μM 3‐isobutyl‐1‐methyl xanthine (IBMX; Sigma-Aldrich), 200 μM indomethacin (Sigma), and 1 μM dexamethasone (Sigma-Aldrich). The chondrogenic differentiation medium was composed of DMEM/High media with 10% FBS, 1% P/S, 1% Insulin‐Transferrin‐Selenium‐A (Gibco), 50 μg/mL ascorbic acid (Sigma-Aldrich), 100 nM dexamethasone, and 10 ng/mL transforming growth factor β1 (TGF‐β1; Peprotech, Cranbury, NJ, USA). Osteogenic and chondrogenic differentiation were induced for 3 weeks, while adipogenic differentiation was induced for 2 weeks. We changed the medium every 2 days. After differentiation, 4% paraformaldehyde (Biosesang) was added for 15 min at room temperature. Cells were stained with Oil red O, Alizarin red, and Alcian blue (Sigma-Aldrich) for 30 min, then observed and imaged under a microscope.

**Supplementary Figure**

**Fig. S1.** Pluripotency characterization of human iPSCs. **A** Representative bright-field image showing the typical colonies morphology of undifferentiated hFs-iPSC 3-1 colonies at day 5 (Scale bar = 100 µm). **B** Representative image of iPSC colonies subjected to alkaline phosphatase (AP) staining (Scale bar = 100 µm). **C** Immunocytochemistry for pluripotency markers (OCT4, NANOG, SOX2, and SSEA4), showing nuclear localization of OCT4/NANOG/SOX2 and membrane staining of SSEA4 (Scale bar = 100 µm).

**Fig. S2.** Characterization and mesodermal-marker dynamics during spontaneous differentiation of hiPSCs. **A** qRT-PCR-based quantification of germ layer marker expression during iPSC differentiation (days 5, 7, and 9). Data are presented as mean ± s.d, n = 9. Statistical significance was set at *****p* <0.0001. **B** Morphological changes in spontaneously differentiating (SD)-hiPSCs at day 7 (Scale bar = 100 µm). **C** Flow-cytometric analysis of CD44 and CD90 in SD-hiPSCs.

**Fig. S3.** Cell adhesion and expansion of BMMSCs on FN-coated microparticles. **A** Cell-adhesion efficiency on FN-coated microparticles of BMMSCs. Data are presented as mean ± s.d, n = 3. Statistical significance was set at ns, not significant. **B** Live/dead staining of BMMSCs cultured with FN-coated microparticles (Day 1, 3, and 5) (scale bar = 100 µm).

**Fig. S4.** *In vitro* multilineage differentiation potential of FN-Dex-iMSCs and FN-Pore-iMSCs. Representative staining after lineage induction of FN-Dex-iMSCs and FN-Pore-iMSCs: adipogenic (Oil Red O), osteogenic (Alizarin Red S), and chondrogenic (Alcian Blue) differentiation (Scale bar = 100 µm).
